# Parenting Styles and Psychosocial Factors of Mother–Child Dyads Participating in the ENDORSE Digital Weight Management Program for Children and Adolescents during the COVID-19 Pandemic

**DOI:** 10.3390/children11010107

**Published:** 2024-01-15

**Authors:** Evi Chatzidaki, Vassiliki Chioti, Lidia Mourtou, Georgia Papavasileiou, Rosa-Anna Kitani, Eleftherios Kalafatis, Kostas Mitsis, Maria Athanasiou, Konstantia Zarkogianni, Konstantina Nikita, Christina Kanaka-Gantenbein, Panagiota Pervanidou

**Affiliations:** 1 First Department of Pediatrics, Medical School, National and Kapodistrian University of Athens, Aghia Sophia Children’s Hospital, 11527 Athens, Greece; evi.s.chatzidaki@gmail.com (E.C.); vchioti@yahoo.com (V.C.); ckanaka@med.uoa.gr (C.K.-G.); 2 Postgraduate Course on “The Science of Stress and Health Promotion”, Medical School, National and Kapodistrian University of Athens, 11527 Athens, Greece; lmourtou@med.uoa.gr (L.M.); georgiapapav@med.uoa.gr (G.P.); rosakitani@med.uoa.gr (R.-A.K.); 3 School of Electrical and Computer Engineering, National Technical University of Athens, 15780 Athens, Greece; leftkal@biosim.ntua.gr (E.K.); kmhtshs@biosim.ntua.gr (K.M.); mathanasiou@biosim.ntua.gr (M.A.); kzarkog@biosim.ntua.gr (K.Z.); knikita@ece.ntua.gr (K.N.); 4 Department of Advanced Computing Sciences, Faculty of Sciences and Engineering, Maastricht University, 6200 MD Maastricht, The Netherlands

**Keywords:** weight management, children, e-health, m-health, parenting styles, psychosocial factors

## Abstract

Childhood obesity is a complex disease with multiple biological and psychosocial risk factors. Recently, novel digital programs were developed with growing evidence for their effectiveness in pediatric weight management studies. The ENDORSE platform consists of mobile applications, wearables, and serious games for the remote management of childhood obesity. The pilot studies included 50 mothers and their children aged 6–14 years and resulted in a clinically significant BMI z-score reduction over 4 to 5 months. This secondary analysis of the ENDORSE study focuses on parenting styles and psychosocial factors. Methodology: Semi-structured clinical interviews were conducted with all participating mothers pre-and post-intervention. The Parenting Styles and Dimensions Questionnaire (PSDQ) evaluated the mothers’ parenting styles. The psychosocial functioning of the participating children was assessed with the parental version of the Strengths and Difficulties Questionnaire (SDQ). The relationship between parenting styles, psychosocial parameters, and weight outcomes was investigated using a linear regression analysis. Results: Weight-related stigma at school (56%), body image concerns (66%), and difficulties in family relationships (48%) were the main concerns documented during the initial psychological interviews. According to the SDQ, there was a significant decrease in children’s conduct problems during the study’s initial phase (pre-pilot group). A decrease in maternal demandingness (i.e., strict parenting style) was associated with a decrease in BMI z-score (beta coefficient = 0.314, *p*-value = 0.003). Conclusion: Decreasing parental demandingness was associated with better weight outcomes, highlighting the importance of assessing parenting factors in pediatric weight management programs.

## 1. Introduction

Obesity is a chronic disease with multiple biological and environmental risk factors [1,2,3,4]. According to the recently published data in the World Obesity Atlas 2023, obesity is expected to increase from 2020 to 2035 in all age groups. The steepest increase is expected among children and adolescents [5]. The recent COVID-19 pandemic has worsened the burden of obesity in adults and children [6,7,8,9,10]. The measures applied by governments around the world to control the pandemic (i.e., lockdowns with school closures, staying in, and social distancing orders) were linked to changes in health behaviors and subsequent weight gain in children [11,12,13,14,15]. Moreover, lockdown measures have disrupted children’s daily routines, which are very important for children’s mental health [16,17,18]. Studies have shown that children’s emotional states during the pandemic were linked to lifestyle changes [18,19]. 

An association between childhood obesity and multiple psychosocial comorbidities like depression, anxiety, eating disorders, poor self-image, poor self-esteem, body dissatisfaction, weight-related stigma, poor quality of life, conduct problems, and peer problems is already established [1,20,21,22,23,24,25,26]. Poor family functioning is also considered a risk factor for excess weight in childhood [27]. Intensive health behavior and lifestyle treatment (IHBLT) programs are considered first-line options for childhood obesity [1]. IHBLT programs are reported to be more effective when they consist of face-to-face meetings, involve all family members, and include lessons about nutrition, physical activity, and behavioral change [1]. A minimum of 26 h over 3 to 12 months is considered important for successful outcomes [1,28]. At the same time, there is growing evidence for the effectiveness of virtual weight management programs [1,29,30,31,32]. No reports exist of adverse psychological impacts of IHBLT programs [28]. On the contrary, recent meta-analyses of age-appropriate multicomponent behavioral weight management programs have shown a positive impact on children’s self-esteem and body image [33], depression, anxiety [34], and risk of eating disorders [35]. 

Parenting style refers to the interactions between a parent and their child and the emotional environment in which these interactions occur [36]. Parenting styles are commonly measured using two underlying dimensions: demandingness and responsiveness/warmth, resulting in four distinct parenting styles: authoritative (high responsiveness and high demandingness), permissive (high responsiveness and low demandingness), authoritarian (low responsiveness and high demandingness), and neglectful (low responsiveness and low demandingness) [37,38,39]. The recently released guidelines of the American Academy of Pediatrics for the management of childhood obesity highlight the important roles of family and home environment factors as influencers of children’s weight status [1]. Evidence from prospective studies shows that authoritative parenting may offer protection against later overweight and obesity, although findings are not conclusive [40]. In the feeding domain, the dimensions of responsiveness and demandingness are used to form the four parenting feeding styles (authoritative, authoritarian, permissive/indulgent, and uninvolved), which refer to the emotional climate a parent creates together with their child during meals [41]. The authoritative feeding style (i.e., where parents respond to the child’s cues of hunger and satiety) is considered protective against excessive weight gain, while the indulgent feeding style (responsive and warm but lenient with rules) is associated with a higher weight status in children [1,42,43]. The authoritarian (restrictive) feeding style, i.e., where parents encourage eating with directive, rule-based demands through parent-centric rules, regardless of child preferences, has not been associated with later child weight status in prospective studies so far [42]. Unlike parenting styles, feeding practices refer to the specific goal-directed behaviors used by parents to directly influence their children’s eating habits [43]. High control in feeding (i.e., restrictive feeding practices) may lead to the development of childhood obesity [43,44]. In contrast, positive parental practices like modeling eating healthy foods [45], monitoring child activities [46], praise [45,47], low parental pressure to eat [48,49], and a decrease in restrictive feeding practices [50] have been linked to successful weight outcomes in family-based interventions.

To the best of our knowledge, associations between parenting feeding styles and weight outcomes during family-based weight management programs have not been published so far, while the role of general parenting dimensions has been investigated in only a few weight management studies [51,52,53]. A higher level of baseline parental warmth was associated with better weight outcomes in one family-based trial study [52], while only two studies investigated the role of changes in parenting dimensions during the course of their interventions [51,53]. One of these studies found a link between an increase in parental warmth during the intervention and better weight outcomes for the children at a one-year follow-up assessment [42], while the other study found a link between increased parental responsiveness and decreased parental demandingness with increased family meals at the end of the intervention [53]. In our previous publication, we showed an association between an increase in positive feeding practices and a decrease in BMI z-score [54]. The objective of this paper is to present a thorough analysis of the psychological assessment of the participants in the ENDORSE study and potential adverse psychological events. Another objective is to investigate whether changes in maternal parenting styles were associated with changes in children’s BMI z-scores (post hoc analysis). Specifically, we propose that changes in parenting toward a less demanding style would result in increased autonomy and better self-regulation in children and subsequently better weight outcomes.

## 2. Methodology

### 2.1. Study Design

**Figure 1:**
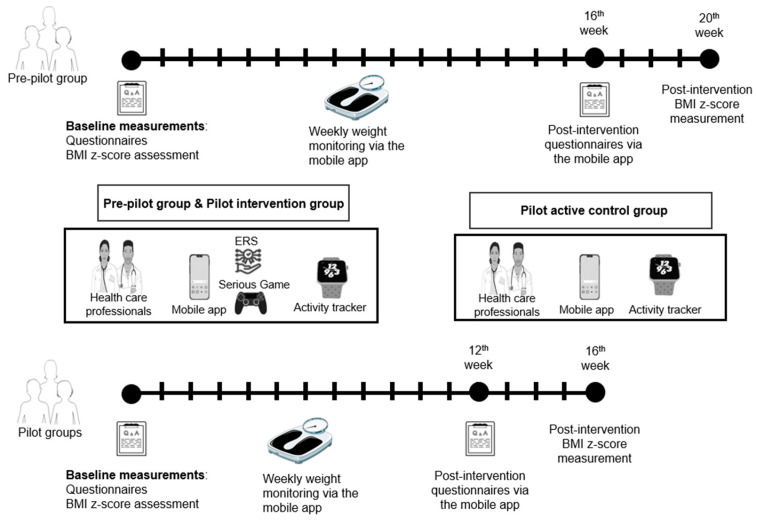
Study timetable and components of the ENDORSE digital platform. The parental mobile application (MA) facilitated daily monitoring of the children’s health behaviors and weekly monitoring of their weight while the MA for health professionals facilitated remote monitoring and communication with the parents. Activity trackers were used to monitor the children’s physical activity and sleep schedule, and the mobile game targeted the education of children about healthy eating and regular exercise. The ENDORSE recommendation system (ERS) collected data from the aforementioned sources and produced individualized content [55]. Parts of the figure were generated using Servier Medical Art (Smart.servier.com, accessed on 3 December 2023) provided by Servier, licensed under a Creative Commons Attribution 3.0 unported license, and flaticon.com (accessed on 3 December 2023).

This study is a secondary analysis of the behavioral and psychological data collected in the ENDORSE feasibility study for pediatric weight management [55]. The clinical studies (pre-pilot and pilot groups) of the ENDORSE program included 50 mothers and their children (52% girls) and resulted in a clinically meaningful BMI z-score reduction (mean BMI z-score reduction: −0.21 ± 0.26, *p*-value < 0.001), as well as improvements in health behaviors, several metabolic factors, and maternal feeding practices. A detailed description of the ENDORSE project and its primary outcomes can be found elsewhere [54,55]. Data collection occurred at baseline and the end of the intervention. The mean interval between the pre-and post-intervention assessments was 5.19 ± 0.66 months for the pre-pilot group and 4.38 ± 1.14 months for the two pilot groups. Figure 1 illustrates the study’s timetable and the main components of the ENDORSE platform. The bioethics committee of the Aghia Sophia Children’s Hospital approved the study on 10 March 2021 (protocol number: 4760). All participating mothers gave written informed consent for themselves and their children’s participation.

The theoretical background of the ENDORSE study was the self-determination theory (SDT) [56] which, in the feeding domain, is expressed using the recent classification of Di Pasquale et Rivolta [57]. Briefly, this classification divides feeding practices into three groups: relatedness—enhancing food parenting practices like family meals and encouraging children’s involvement in preparing meals; competence—enhancing food parenting practices like clear and consistent rules related to food, the availability of healthy food at home, educating children about nutrition, and parental modeling; and autonomy—enhancing food parenting practices like guided choices and discussing food choices with children choices. Messages (daily and weekly messages) that were sent to mothers via the application were written according to these principles. For example, one of the daily messages sent by the Endorse Recommendation System to the mothers was “Arrange family meals as often as possible”. Moreover, these practices were explained to the mothers during a 90 min face-to-face session at the beginning of the study. Also, at that time, all participants received that time a printed educational booklet with advice about healthy eating which included a brief description of these feeding practices. The educational booklet was also available in a PDF version via the mobile application, as described fully in our previous publications [54,55]. Moreover, the mothers were responsible for the daily monitoring of their children’s health behavior goals. Briefly, six health behavior goals changed every 2 weeks: one double goal, i.e., physical activity goal and screen time goal for the first 2 weeks of the study, and, subsequently, five dietary goals based on the personalized dietary plan of each child (i.e., breakfast, mid-morning snack, lunch, afternoon snack, and dinner goals). The five dietary goals were also designed according to the SDT principles. For example, mothers were advised not to pressure their children to have breakfast in case they did not want to but instead to prepare a healthy mid-morning snack for their children to take with them to school. Also, mothers were advised to offer choices to their children at each meal, while children were advised to eat until full during the main meals to promote self-regulation. A detailed description of all health behavior goals can be found elsewhere [55]. The aforementioned practices are possibly related to decreasing parental demandingness and increasing children’s autonomy in the feeding domain.

### 2.2. Participants and Settings

This study took place from March 2021 to May 2022, and participant recruitment was conducted from the Obesity Outpatient Clinic of the First Department of Pediatrics of the National and Kapodistrian University of Athens at the Aghia Sophia Children’s Hospital, in Athens, Greece. Children and adolescents aged 6–14 years old with a body mass index (BMI) > 85th centile for age and sex were eligible for the study. The exclusion criteria were secondary causes of excess weight (i.e., endocrine causes, genetic syndromes linked with overweight and obesity, and serious developmental disorders).

The first phase of the ENDORSE study (pre-pilot group) began in March 2021, included 20 children with their mothers, and was completed in September 2021. The attrition rate of the pre-pilot group was 10% (2 children). The second phase (pilot group) began in September 2021, included 30 children with their mothers, and was completed in May 2022. The attrition rate of the pilot group was 10% (3 children). The pilot groups were divided into an active control group and an intervention group, both consisting of 15 mother–child dyads (Figure 1). Details on the 3 consecutive groups of the ENDORSE study can be found elsewhere [55].

### 2.3. Measures

#### 2.3.1. Clinical and Nutritional Assessments

At the beginning and the end of the study, children underwent a clinical evaluation by a pediatric endocrinologist. The children’s weight, height, and pubertal stage were assessed using standardized methods as described elsewhere [54]. The weight status of the children was divided into three groups according to International Obesity Force (IOTF) criteria [58]. Overweight was defined as BMI > 85th centile, obesity as BMI > 95th centile, and severe obesity as BMI ≥ 120% of the 95th centile for age and sex [58,59]. To standardize each BMI, a conversion to a z-score for age and sex was carried out according to the guidelines of the Centers for Disease Control and Prevention (CDC) growth charts 2000 [60]. Further, conversion to adjusted BMI z-scores was performed in children with a BMI > 97th centile, as BMI z-scores are not considered accurate at these values [61]. Moreover, at baseline, the team’s nutritionist performed a thorough nutritional assessment. For all children, a flexible, individualized dietary plan was designed according to the Greek Guidelines of the Mediterranean Dietary Pattern [62], as described in detail elsewhere [55].

#### 2.3.2. Psychological Assessment

At baseline, the team’s psychologist performed a thorough psychological assessment via clinical interviews, which were conducted in person (n = 32, 64%) or by telephone (n = 18, 36%). Mothers and children younger than 12 years were interviewed simultaneously, while adolescents older than 12 years were interviewed separately. Structured open-ended questions were used to assess children’s psychosocial functioning, such as peer relationships/friends, history of weight stigma, body image concerns, and family relationships. In addition, emphasis was given to avoiding unhealthy weight control behaviors (i.e., vomiting, meal skipping, purging, fasting, and excessive exercise). According to the American Academy of Pediatrics, children should not lose more than one kilogram per week [63] and, to this end, parents were advised to monitor their children’s weight once a week to achieve gradual weight loss. A second psychological assessment was carried out at the end of the study and was also conducted in person (n = 15, 33%) or by telephone (n = 30, 67%). The second assessment was brief and focused on potential psychological side effects and the participants’ overall satisfaction or dissatisfaction with the study.

Several questionnaires were completed by the mothers via the application. General socio-demographic information (e.g., maternal marital status, educational level, and occupation) was collected through a questionnaire that was designed by the clinical team (details can be found elsewhere [55]). Maternal parenting styles were assessed using the Parenting Styles and Dimensions Questionnaire (PSDQ) [64]. This questionnaire includes 29 questions and uses a five-point Likert scale. The PSDQ was validated for Greek mothers by Antonopoulou and Tsitsas with very high test–retest reliability (correlation coefficient r = 0.83, *p* < 0.001). While Robinson’s original questionnaire includes three parenting types (supportive, permissive, and authoritarian), the validation in Greek mothers also highlighted a fourth type, the strict mother, which has characteristics of both the supportive (caring for her child’s wishes and feelings) and authoritarian (exercises strict control and sets clear limits on her child’s behavior) types [65]. This questionnaire was also completed by the mothers at the end of the intervention and was used as an indicator of the potential impact this study had on maternal parenting styles.

Mothers also completed the Strengths and Difficulties Questionnaire (SDQ), which was used to assess their children’s behavioral and emotional problems [66]. This questionnaire consists of 25 questions with 3 possible answers (not applicable, somewhat applicable, and applicable) which detect difficulties as well as the potential strengths of children. The 5 areas the SDQ examines are emotional problems, conduct problems, hyperactivity/inattention, peer problems, and prosocial behavior. The Greek version of the questionnaire was used for the study [67]. The SDQ scores were calculated based on the instructions from the SDQ webpage [68]. Scores range from 0 to 10 for all subscales, while the total difficulties score ranges from 0 to 40, based on all but the prosocial scale. SDQ total scores of 17 and above are considered abnormal. Clinical cut-off raw scores for the subscales (out of a possible 10) are emotional problems ≥ 5, conduct problems ≥ 4, hyperactivity ≥ 7, peer relationships ≥ 4, and prosocial behavior ≤ 4. In our primary analysis, we presented the baseline groups of the SDQ results [54], whereas in this secondary analysis, we present the changes in the SDQ scores as an indicator of the potential impact the study had on the overall psychosocial functioning of the children.

### 2.4. Statistical Analysis

Quantitative variables are presented as mean ± standard deviation (SD) values or medians with the 25th and 75th centiles, whereas the qualitative variables are presented as absolute values with percentages. To examine the relationship between categorical variables, the Chi-square test was used. The independent-samples T-test and the Mann–Whitney U test were used for the comparison of normally and non-normally distributed variables, respectively. To investigate possible differences within the participant groups pre- and post-intervention, a paired-samples T-test was performed for normally distributed variables, and the Wilcoxon test was used for non-normally distributed variables. To calculate change variables, baseline variables were subtracted from the post-intervention variables. A power calculation, based on the UCSF sample size calculator [69], was conducted for the primary analysis of our study (i.e., pre- and post-BMI z-score changes), indicating an adequate sample size equal to 34, as presented in detail in our previous publication [55]. Additionally, power calculations were also performed in a post-hoc manner with the UCSF sample size calculator within samples and the effect size calculator between pre-pilot and pilot samples. Additional power analysis information is presented in the footnotes of the corresponding tables for statistically significant results.

A post-hoc analysis was performed to investigate potential associations between changes in parenting styles and changes in BMI z-scores. Initially, the change variables of the four parenting styles (authoritative, strict, permissive, and authoritarian) were entered in bivariate regression models with changes in BMI z-scores as the dependent variable. A multivariate regression analysis followed, using variables that were significantly associated. The children’s covariates were sex (variable values: male = 0, female = 1), age (in years), and BMI z-score at baseline (continuous variable). Maternal covariates included educational level (variable values: primary or secondary educational level = 0, tertiary educational level = 1), and marital status (variable values: unmarried = 0, married or living with a significant other = 1). The regression analysis also included the level of adherence to the study protocol (variable values: medium/low adherence = 0, high adherence = 1) as defined in the paper by Zarkogianni et al. [55], and the baseline value of parenting style (continuous variable) for control purposes. Lastly, to account for the difference in the duration of the pre-pilot and pilot studies (16 weeks versus 12 weeks), the binary variable “group” was created, assuming values of 0 for children participating in the pre-pilot study and 1 for children participating in the pilot study. The alpha value was set at <0.05. A Bonferonni correction was applied at the considered significance level to account for the number of comparisons performed (0.05/9 = 0.0055). The adjusted R-squared estimates were considered for assessing the models’ goodness-of-fit due to the small number of subjects per variable (SPV = 4.56) observed in our sample, as suggested in Austin et al. [70]. The statistical analysis was executed using Python [71].

## 3. Results

### 3.1. Baseline Characteristics

In Table 1, the baseline characteristics of the participating children are presented. Fifty children (52% girls, 58% pubertal) were included in the analysis. Forty-two percent of the children were classified as having severe obesity according to the IOTF criteria [59]. The baseline BMI z-scores of the children between the two groups were similar. Overall, 56 percent of the children experienced weight stigma at school, while 66% had concerns about their body image. According to the parental version of the SDQ, there were no baseline differences in the psychosocial functioning of the children between the two groups.

Baseline maternal characteristics are presented in Table 2. The vast majority of the mothers were Greek (96%), married (78%), and employed (78%). Most of the mothers had an authoritative parenting style, followed by a strict parenting style. At the time of the psychological interview, almost half of the mothers (48%) were concerned about family relationships, mainly focusing on difficulties of mother–child interactions (22%).

### 3.2. Changes in Parenting Styles

Table 3 shows changes in maternal parenting styles at the end of the intervention. Overall, no statistically significant changes in parenting styles within groups or between groups were observed.

### 3.3. Changes in Psychosocial Functioning

In Table 4, changes in the children’s psychosocial functioning at the end of the intervention are presented. Overall, there were no statistically significant changes in psychosocial functioning. Within the groups, a statistically significant decrease in conduct problems in the pre-pilot group (−0.69, *p* = 0.005) was found. A difference was observed between the two groups in conduct problems (a decrease in the pre-pilot group versus an increase in the pilot group, *p* = 0.002). Furthermore, at the brief post-intervention psychological assessment, all mothers expressed their overall satisfaction with their participation in the program, while no concerns were raised about the occurrence of any adverse psychological effects (e.g., the development of unhealthy weight control practices, more family distress).

### 3.4. Associations between Parenting Styles and BMI z-Scores

To investigate whether there was a possible association between the change variables of the four parenting styles and the BMI z-score change, a bivariate analysis was performed. Only a strict parenting style change presented a statistically significant association with a BMI z-score change (beta coefficient = 0.396, *p* = 0.01). Therefore, it was entered into a multivariate regression model. Table 5 shows the results of the multivariate regression analysis. A decrease in strict parenting style was significantly correlated with a decrease in BMI z-score (beta coefficient = 0.468, *p* = 0.003), after adjusting for the child’s age, sex, baseline BMI z-score, maternal education, maternal marital status, baseline strict parenting style, group, and adherence to the study protocol. The total model accounted for 35.8% of the variance in BMI z-score changes (adjusted R squared = 0.358, F-statistic: 3.475, *p* = 0.005). The complete models can be found in the Supplementary Materials.

## 4. Discussion

The pilot studies of the ENDORSE weight management program included 50 mother–child dyads and resulted in a clinically and statistically significant BMI z-score reduction [55]. The focus of this secondary analysis is on parenting styles and the psychosocial factors of mother–child dyads. The main concerns that were raised during the initial psychological interviews were body image dissatisfaction, weight-related stigma at school, and difficulties in family relationships. These findings are in accordance with many studies that have been published so far and have documented higher levels of body image concerns and weight-related stigma in children with elevated BMI status in comparison to children with normal weight [24,25,33,72]. Poor family functioning is also known to be related to a higher risk of obesity in children and adolescents; specifically, poor communication, poor behavior control, high levels of family conflict, and low family hierarchy values are aspects that have been associated with a higher risk of excess weight in children [27].

During the brief post-intervention psychological interview with the mothers, overall satisfaction with the ENDORSE program was documented, while no adverse psychological events were reported. This is in line with other studies that report no adverse psychological outcomes in face-to-face, and in digital weight management programs for childhood obesity [28,30]. Overall, no statistically significant changes in children’s emotional and behavioral aspects were documented pre-and post-intervention. Specifically in the pre-pilot group, a statistically significant decrease in conduct problems was documented, and between groups, differences were observed in the changes in conduct problems (a decrease in the pre-pilot group versus a small increase in the pilot group). The SDQ is among the most commonly used questionnaires assessing mental health in children [73,74,75]. It is well documented in the literature that excess weight is linked to higher incidences of internal (i.e., emotional problems) and external problems (i.e., conduct problems or hyperactivity) in children and adolescents [20,21,22,23].

An important consideration when interpreting the differences in children’s psychological functioning between participants in the pre-pilot phase and the pilot phase of the study is the social circumstances under which these phases of the ENDORSE program took place. The pre-pilot study started in March 2021 and was completed in September 2021, a period that coincided with the evolution of the third wave of the COVID-19 pandemic in Greece [76], whereas the pilot study started in September 2021 and was completed in May 2022, a period that coincided with the evolution of milder Omicron variants of SARS-CoV-2 and higher vaccination rates in Greece [77]. At the time of the pre-intervention assessment of the pre-pilot group, Greece was in national lockdown due to the government’s measures to control the pandemic, with strict social distancing rules, schools closed, and the suspension of sports and recreational activities [76]. Many studies have found that strict lockdown measures which cause dramatic lifestyle changes have a serious impact on the psychological well-being of children regardless of weight status [19,78]. On the contrary, at the time of the pilot study, schools remained open, and the measures to control the pandemic were less strict in Greece compared to the measures during the time of the pre-pilot study [77]. This difference could partially explain the greater decrease in conduct problems reported by mothers during the pre-pilot study.

The strict parenting style, i.e., a combination of authoritative and authoritarian parenting styles, is unique to Greek mothers [65], and its potential associations with weight outcomes in children have not been investigated so far. In our study, a decrease in maternal demandingness was associated with a decrease in BMI z-score. Although we did not explicitly target parenting styles in the ENDORSE study, we encouraged mothers to avoid strict limitations according to the principles of the self-determination theory [56,57] and the principles of positive parenting in the feeding domain [39]. Participating mothers were educated in practices possibly related to decreased demandingness, such as autonomy-supportive food parenting practices [57] like negotiating rules and offering choices to children, and relatedness-promoting feeding practices [57] like having frequent family meals and encouraging the participation of children in meal preparation [54,55]. Moreover, for every participating child, a flexible, individualized dietary plan was designed according to these principles, with every meal consisting of 2–3 different meal options. Furthermore, children were encouraged to eat until full during main meals to promote self-regulation [55]. We believe that the aforementioned practices led to a less demanding parenting style in the feeding domain and subsequently increased autonomy and self-regulation in children, which is possibly related to their better weight outcomes. Nevertheless, an assessment of feeding styles would have been more appropriate in this context. Unfortunately, currently, there is no validated feeding-style questionnaire available in Greece.

To the best of our knowledge, two face-to-face studies have been published so far which show that the parental dimension of warmth is associated with better weight outcomes in children [51,52]. A study by Rhee et al., which included forty families in a 16-week family-based program for weight control, found that a higher level of warmth measured by the General Parenting Observational Scale was associated with increased odds of a decreasing/stable child BMI immediately after the program [52]. Another family-based weight management program by Stein et al., which included fifty children with excess weight, demonstrated that an increase in children’s perceived paternal acceptance after treatment, was associated with better changes in percentage overweight over 12 months in comparison to children with lower ratings of paternal acceptance [51]. A more recent study of 242 parent–child dyads integrated cultural tailoring, parenting, behavioral, and motivational strategies to address African American adolescent weight loss [79]. Although no significant effects on BMI were observed immediately post-intervention [79], the authors demonstrated that increased parental responsiveness and reduced parental demandingness were associated with an increased frequency of family mealtime [53]. These results highlight the fact that more research is required in large, culturally diverse populations to clarify the role of parenting style and its interactions with feeding styles, feeding practices, and children’s eating behaviors in pediatric weight management trials.

## 5. Conclusions and Limitations

The ENDORSE program took place during the COVID-19 pandemic. Restricted clinic visit policies were among the public measures applied by the Greek government to control the spread of SARS-CoV-2, which did not allow for the recruitment of a larger sample and subsequently led to the absence of a control group. Thus, our results cannot be generalized. Moreover, our sample was not diverse as it consisted of treatment-seeking mother–child dyads. A further limitation is that only mothers participated, and no other family members were included in the study. Moreover, we only administered the parental version of the Strengths and Difficulties Questionnaire, while we included older children (>11 years) to whom we could have administered the self-reported version. Lastly, the range of children’s ages included in our study was wide (6–14 years). Younger age displayed some association with a BMI z-score reduction in our regression model; however, no definite conclusion can be drawn from this particular study since statistical significance was not displayed. Therefore, in future studies, further analysis of the results according to children’s age could provide useful insights on its impact on children’s weight outcomes. Moreover, in the design of future studies, larger, more diverse samples should be included, with a control group. Despite these limitations, in the ENDORSE project, decreasing parental demandingness was associated with better weight outcomes in children with excess weight. This interesting finding could inform future studies that could focus primarily on changing parenting styles and then examine the impact of parent training on the weight outcomes of their children.

## Figures and Tables

**Table 1:** Baseline children’s characteristics.

Characteristics	Total (n = 50)	Pre-Pilot Group (n = 20)	Pilot Group (n = 30)	*p*-Value (between Groups)
Age	10.49 (1.99)	10.94 (1.85)	10.19 (2.05)	0.193
Sex: Female Male	26 (52) 24 (48)	8 (40) 12 (60)	18 (60) 12 (40)	0.248
Pubertal stage: Prepubertal Pubertal	21 (42) 29 (58)	8 (40) 12 (60)	13 (43.3) 17 (56.7)	1.00
Weight	69.82 (22.53)	76.94 (22.48)	65.08 (21.64)	0.068
Height	1.49 (0.13)	1.51 (0.13)	1.48 (0.14)	0.349
BMI	30.65 (6.22)	33.02 (6.51)	29.06 (5.58)	0.026 ^1^
BMI z-score	2.85 (2.23, 4.07)	2.85 (2.57, 4.38)	2.79 (1.95, 3.47)	0.104
Weight Status: (IOTF criteria) [59] Overweight Simple obesity Severe obesity	4 (8) 25 (50) 21 (42)	0 11 (55) 9 (45)	4 (13.3) 14 (46.7) 12 (40)	0.234
Weight stigma experience at school: ^1^ Yes No	28 (56) 22 (44)	11 (55) 9 (45)	17 (56.7) 13 (43.3)	1.00
Body image concerns: ^1^ Yes No	33 (66) 17 (34)	12 (60) 8 (40)	21 (70) 9 (30)	0.548
SDQ scales in scores: Total difficulties Emotional problems Conduct problems Hyperactivity Peer relationships Prosocial behavior	10.66 (6.16) 3.10 (2.39) 2.62 (1.61) 3.42 (2.50) 1.52 (1.57) 8.82 (1.21)	10.95 (6.24) 2.75 (2.15) 2.90 (1.83) 3.90 (2.79) 1.40 (1.39) 8.40 (1.27)	10.47 (6.21) 3.33 (2.55) 2.43 (1.45) 3.10 (2.28) 1.60 (1.69) 9.10 (1.09)	0.789 0.404 0.322 0.272 0.663 0.043 ^2^

Continuous variables are presented as mean (SD) or median (25th and 75th percentiles) values, whereas categorical variables are presented as absolute numbers (n) and frequencies (%). ΒΜΙ: body mass index, IOTF: International Obesity Task Force, SDQ: Strengths and Difficulties Questionnaire. ^1^ assessed by clinical interview (psychologist). ^2^ insufficiently powered.

**Table 2:** Baseline maternal characteristics.

Characteristics	Total (n = 50)	Pre-Pilot Group (n = 20)	Pilot Group (n = 30)	*p*-Value (between Groups)
Age	43.90 (5.34)	44.35 (5.08)	43.6 (5.57)	0.632
BMI	30.01 (6.07)	30.71 (6.35)	29.55 (5.94)	0.514
Ethnicity: Greek Albanian Romanian	48 (96) 1 (2) 1 (2)	19 (95) 0 1 (5)	29 (96.7) 1 (3.3) 0	0.338
Marital status: Married Single Divorced Widowed	39 (78) 3 (6) 7 (14) 1 (2)	14 (70) 1 (5) 4 (20) 1 (5)	25 (83.3) 1 (3.3) 4 (13.3) 0	0.413
Educational level: Primary Secondary Tertiary	4 (8) 27 (54) 19 (38)	3 (15) 12 (60) 5 (25)	1 (3.3) 15 (50) 14 (46.7)	0.154
Occupation: Employed Unemployed	39 (78) 11 (22)	14 (70) 6 (30)	25 (83.3) 5 (16.7)	0.311
Maternal annual income: EUR <10,000 EUR 10,000–20,000 EUR >200,000 Omission	12 (24) 12 (24) 4 (8) 21 (42)	7 (35) 7 (35) 1 (5) 5 (25)	5 (16.7) 5 (16.7) 3 (10) 16 (53.3)	0.113
Parenting style (PSDQ): (5-point Likert scale) Authoritative Strict Permissive Authoritarian	4.27 (0.47) 3.56 (0.55) 2.86 (0.77) 1.61 (0.58)	4.28 (0.55) 3.46 (0.47) 2.81 (0.89) 1.57 (0.57)	4.26 (0.42) 3.62 (0.59) 2.89 (0.70) 1.64 (0.60)	0.924 0.334 0.735 0.681
Family functioning: ^1^ No concerns Mother–child difficulties Sibling rivalry/teasing Multi-level difficulties (parents and siblings)	26 (52) 11 (22) 8 (16) 5 (10)	13 (65) 4 (20) 1 (5) 2 (10)	13 (43.3) 7 (23.3) 7 (23.3) 3 (10)	0.300
Number of children: One child Two children Three children Four or five children	11 (22) 30 (60) 4 (8) 5 (10)	6 (30) 10 (50) 1 (5) 3 (15)	5 (16.7) 20 (66.7) 3 (10) 2 (6.7)	0.435

Continuous variables are presented as mean (SD) or median (25th and 75th percentiles) values, whereas categorical variables are presented as absolute numbers (n) and frequencies. ΒΜΙ: Body Mass Index, PSDQ: Parenting Styles and Dimensions Questionnaire. ^1^ assessed by clinical interview (psychologist).

**Table 3:** Changes in maternal parenting styles pre-and post-intervention.

	Total (n = 43)	Pre-Pilot Group (n = 16)	Pilot Group (n = 27)	*p*-Value (between Groups)
Authoritative	0.02 (0.31)	0.07 (0.22)	−0.003 (0.35)	0.961
Strict	−0.05 (0.38)	−0.06 (0.40)	−0.04 (0.36)	0.513
Permissive	0.07 (0.61)	0.06 (0.53)	0.08 (0.66)	0.140
Authoritarian	−0.02 (0.36)	0.05 (0.22)	−0.06 (0.42)	0.060

Values are presented as mean (SD) values. All *p* values within groups were non-significant (*p* > 0.05).

**Table 4:** Changes in children’s behavioral and emotional aspects evaluated by changes in SDQ scores (parental version) [66].

SDQ Scores	Total (n = 43)	Pre-Pilot Group (n = 16)	Pilot Group (n = 27)	*p*-Value (between Groups)
Total difficulties	0.74 (4.76)	0.19 (4.26)	1.07 (5.08)	0.553
Emotional problems	0.28 (2.13)	0.06 (2.21)	0.41 (2.12)	0.838
Conduct problems	0.02 (1.16)	**−0.69** ^1^ (0.70)	0.44 (1.19)	**0.002** ^2^
Hyperactivity/ inattention	0.35 (2.06)	0.69 (1.85)	0.15 (2.18)	0.407
Peer problems	0.33 (1.38)	0.56 (1.46)	0.19 (1.33)	0.262
Prosocial behavior	0.12 (1.18)	0.50 (1.10)	−0.11 (1.19)	0.150

Values are presented as mean (SD) values. ^1^ *p* value = 0.005 within group, sufficiently powered (minimum required dyads equal to 11); ^2^ sufficiently powered, minimum detected effect size equal to 1.05 (in our case, 1.25). Statistically significant differences are presented in bold.

**Table 5:** Linear regression model results.

Independent Variables	Dependent Variable: BMI z-Score Change (n = 41)		
	Beta	SE	*p*-Value
Child age	0.315	0.019	0.052
Child sex	−0.014	0.074	0.924
Child baseline BMI z-score	−0.010	0.027	0.946
Maternal marital status	0.274	0.080	0.044
Maternal education	0.331	0.076	**0.033**
Baseline strict parenting style	−0.015	0.065	0.918
Strict parenting style change	0.468	0.098	0.003
Group	0.107	0.075	0.464
Adherence	−0.307	0.071	0.032

Beta: standardized beta coefficient, SE: coefficient of standard error. Statistically significant differences are presented in bold.

## Data Availability

The data are not publicly available due to privacy restrictions.

## Supplementary Materials

The following supporting information can be downloaded at: https://www.mdpi.com/article/10.3390/children11010107/s1, Linear Regression Models Results.
