# Supplementary material for: Parenting Styles and Psychosocial Factors of Mother–Child Dyads Participating in the ENDORSE Digital Weight Management Program for Children and Adolescents during the COVID-19 Pandemic
Source: Children (Basel). 2024 Jan 15;11(1):107. doi: 10.3390/children11010107 (PMC10814028; doi:10.3390/children11010107)
Supplement: Supplementary file 1 [file children-11-00107-s001.zip › children-2789542-supplementary.pdf]

Linear Regression Model Results

Dependent variable: BMI z-score change

Predictor variables: Sex, age, group, baseline BMI z-score, marital status, maternal education, baseline strict parenting style, strict parenting style change, adherence

**Dep. Variable:**	**BMI z-Score Change**	**R-squared:**	**0.502**
Model:	OLS	Adj. R-squared:	0.358
Method:	Least Squares	F-statistic:	3.475
Date:	Wed, 22 Nov 2023	Prob (F-statistic):	0.00453
Time:	12:53:47	Log-Likelihood:	12.631
No. Observations:	41	AIC:	−5.263
Df Residuals:	31	BIC:	11.87
Df Model:	9		
Covariance Type:	nonrobust		

Summary Table

	**coef**	**Beta**	**std err**	**t**	**P>|t|**	**[0.025**	**0.975]**
Intercept	-0.6558	−2.5703	0.323	−2.031	0.051	−1.314	0.003
Sex	-0.0072	−0.0143	0.074	−0.096	0.924	−0.159	0.145
Age	0.0392	0.3154	0.019	2.023	0.052	-0.000	0.079
Group	0.0558	0.1066	0.075	0.742	0.464	-0.097	0.209
Baseline BMI z-score	-0.0018	−0.0099	0.027	−0.068	0.946	−0.057	0.053
Marital status	0.1669	0.2741	0.080	2.098	0.044	0.005	0.329
Maternal educational status	0.1691	0.3306	0.076	2.238	0.033	0.015	0.323
Baseline strict parenting style	−0.0068	−0.0152	0.065	-0.104	0.918	−0.140	0.126
Strict parenting style change	0.3144	0.4683	0.098	3.225	0.003	0.116	0.513
Adherence	-0.1606	−0.3069	0.071	−2.252	0.032	−0.306	−0.015

Linear Regression Model Results

Dependent variable: BMI z-score change

Predictor variables: Strict parenting style change

**Dep. Variable:**	**BMI z-score change**	**R-squared:**	**0.157**
Model:	OLS	Adj. R-squared:	0.135
Method:	Least Squares	F-statistic:	7.258
Date:	Wed, 22 Nov 2023	Prob (F-statistic):	0.0104
Time:	12:53:47	Log-Likelihood:	1.8307
No. Observations:	41	AIC:	0.3387
Df Residuals:	39	BIC:	3.766
Df Model:	1		
Covariance Type:	nonrobust		

Summary Table

	coef	Beta	std err	t	P>|t|	[0.025	0.975]
Intercept	−0.1951	−0.7647	0.037	−5.221	0.000	−0.271	−0.119
Strict parenting style change	0.2659	0.3961	0.099	2.694	0.010	0.066	0.466

Linear Regression Model Results

Dependent variable: BMI z-score change

Predictor variables: Authoritarian parenting style change

**Dep. Variable:**	**BMI z-Score Change**	**R-squared:**	**0.068**
Model:	OLS	Adj. R-squared:	0.044
Method:	Least Squares	F-statistic:	2.825
Date:	Wed, 22 Nov 2023	Prob (F-statistic):	0.101
Time:	12:53:47	Log-Likelihood:	−0.23426
No. Observations:	41	AIC:	4.469
Df Residuals:	39	BIC:	7.896
Df Model:	1		
Covariance Type:	nonrobust		

Summary Table

	coef	Beta	std err	t	P>|t|	[0.025	0.975]
Intercept	−0.2062	−0.8082	0.039	−5.288	0.000	−0.285	−0.127
Authoritarian parenting style change	0.1798	0.2600	0.107	1.681	0.101	−0.037	0.396

Linear Regression Model Results

Dependent variable: BMI z-score change

Predictor variables: Authoritative parenting style change

**Dep. Variable:**	**BMI z-score change**	**R-squared:**	**0.001**
Model:	OLS	Adj. R-squared:	−0.025
Method:	Least Squares	F-statistic:	0.02344
Date:	Wed, 22 Nov 2023	Prob (F-statistic):	0.879
Time:	12:53:47	Log-Likelihood:	−1.6558
No. Observations:	41	AIC:	7.312
Df Residuals:	39	BIC:	10.74
Df Model:	1		
Covariance Type:	nonrobust		

Summary Table

	coef	Beta	std err	t	P>|t|	[0.025	0.975]
Intercept	−0.2078	−0.8144	0.040	−5.145	0.000	−0.289	−0.126
Authoritative parenting style change	−0.0207	−0.0245	0.135	−0.153	0.879	−0.295	0.253

Linear Regression Model Results

Dependent variable: BMI z-score change

Predictor variables: Permissive parenting style change

**Dep. Variable:**	**BMI z-Score Change**	**R-squared:**	**0.009**
Model:	OLS	Adj. R-squared:	−0.016
Method:	Least Squares	F-statistic:	0.3537
Date:	Wed, 22 Nov 2023	Prob (F-statistic):	0.555
Time:	12:53:47	Log-Likelihood:	−1.4830
No. Observations:	41	AIC:	6.966
Df Residuals:	39	BIC:	10.39
Df Model:	1		
Covariance Type:	nonrobust		

Summary Table

	**coef**	**Beta**	**std err**	**t**	**P>|t|**	**[0.025**	**0.975]**
Intercept	−0.2028	−0.7948	0.041	−4.933	0.000	−0.286	−0.120
Permissive parenting style change	−0.0424	−0.0949	0.071	−0.595	0.555	−0.186	0.102
